# Cirrhotic Liver of Liver Transplant Recipients Accumulate Silver and Co-Accumulate Copper

**DOI:** 10.3390/ijms22041782

**Published:** 2021-02-11

**Authors:** Jarosław Poznański, Dariusz Sołdacki, Bożena Czarkowska-Pączek, Arkadiusz Bonna, Oskar Kornasiewicz, Marek Krawczyk, Wojciech Bal, Leszek Pączek

**Affiliations:** 1 Institute of Biochemistry and Biophysics, Polish Academy of Sciences, Pawińskiego 5a, 02-106 Warsaw, Poland; jarek@ibb.waw.pl; 2 Department of Clinical Immunology, Medical University of Warsaw, Nowogrodzka 59, 02-006 Warsaw, Poland; dariusz.soldacki@wum.edu.pl; 3 Department of Clinical Nursing, Medical University of Warsaw, E. Ciołka 27, 01-445 Warsaw, Poland; bozena.czarkowska-paczek@wum.edu.pl; 4 Department of Biochemistry, University of Cambridge, Tennis Court Road, Downing Site, Cambridge CB2 1QW, UK; ab2295@cam.ac.uk; 5 Department of General, Transplant and Liver Surgery, Medical University of Warsaw, Banacha 1a, 02-097 Warsaw, Poland; oskar.kornasiewicz@wum.edu.pl (O.K.); marek.krawczyk@wum.edu.pl (M.K.); 6 Department of Immunology, Transplantology and Internal Diseases, Medical University of Warsaw, Nowogrodzka 59, 02-006 Warsaw, Poland

**Keywords:** liver disease, liver transplant, copper, silver

## Abstract

Silver-based materials are widely used in clinical medicine. Furthermore, the usage of silver containing materials and devices is widely recommended and clinically approved. The impact on human health of the increasing use of silver nanoparticles in medical devices remains understudied, even though Ag-containing dressings are known to release silver into the bloodstream. In this study, we detected a widespread and sometimes significant silver accumulation both in healthy and sick liver biopsies, levels being statistically higher in patients with various hepatic pathologies. 28 healthy and 44 cirrhotic liver samples were investigated. The median amount of 0.049 ppm Ag in livers was measured in cirrhotic livers while the median was 0.0016 ppm for healthy livers (a more than 30-fold difference). The mean tissue concentrations of essential metals, Fe and Zn in cirrhotic livers did not differ substantially from healthy livers, while Cu was positively correlated with Ag. The serum levels of gamma-glutamyl transpeptidase (GGTP) was also positively correlated with Ag in cirrhotic livers. The increased Ag accumulation in cirrhotic livers could be a side effect of wide application of silver in clinical settings. As recent studies indicated a significant toxicity of silver nanoparticles for human cells, the above observation could be of high importance for the public health.

## 1. Introduction

Nanotechnology is currently engaged in developing advanced functional materials for industrial and biomedical applications. Special attention is paid to metal nanoparticles, in particular silver nanoparticles (AgNPs) due to their unique properties including electrical conductivity, chemical stability, and antifungal and antibacterial properties. AgNPs are widely use in the health care system, particularly in antibacterial and antifungal coatings, water purification, medical devices, cancer therapies, and air quality management [1,2]. Some examples of the use of silver include second-generation catheters impregnated with chlorhexidine and silver sulfadiazine, which are more effective in reducing the catheter colonization and infection. AgNPs are also a suitable candidate for the antimicrobial coatings in other medical devices [3]. Silver is applied as a component of bone scaffolds due to the enhancement of their mechanical properties for bone tissue engineering applications [4]. Oral health could benefit from new generation of bioactive resins containing AgNPs [5,6]. In addition to their broad usage in dressings due to their biocidal properties, silver products indirectly reduce odor [7]. AgNPs are also used in other consumer products, for example, in textiles, cosmetics, food packing films, and electronic devices. The mechanism of biocidal properties of AgNPs still remains unclear. It has been proposed to stem from the direct contact of nanoparticles with the bacterial wall, resulting in its damage followed by cell death and/or from the release of Ag(I) ions [8]. The latter results in adventitious Ag(I) binding to multiple bacterial proteins which compromises their function [9]. AgNPs are made of the Ag(0) core that is oxidized at the surface into Ag(I) ions with subsequent distribution of Ag(I) species throughout the cell mainly in the form of Ag–thiol complexes [10,11,12,13]. This type of complexation comes as no surprise as Ag has a picomolar or higher affinity for thiols depending on the coordination chemistry offered by the biomolecular ligands [14,15].

The increasing production of AgNPs has led to environmental and human safety concerns over the past few years. AgNPs could be released into water where they are oxidized into Ag^+^ ions that are toxic and thereby poisonous to plants and aquatic life. Moreover, they gain access to human daily life by entering the food chain. In humans, the main and extensively described exposure route for Ag is dietary, but it could be also inhalation or dermal contact [16]. In vitro and in vivo studies confirmed the dose-, time-, and size-dependent cytotoxicity of AgNPs to several human cell lines including human bronchial epithelial cells, human umbilical vein endothelial cells, skin keratinocytes, human dermal fibroblasts, human hepatocellular liver carcinoma, and others. The mechanism of cytotoxicity is complex and includes activation of endoplasmatic reticulum stress signaling pathway, intracellular ROS formation, reduction of cell proliferation, mitochondrial dysfunction, cell membrane damage, and induction of apoptosis [1]. In brain glial cells, AgNPs induce production of proinflammatory cytokines, reactive oxygen species, and nitric oxide, resulting in the neuroinflammation [17]. Animal studies confirmed results obtained from in vitro studies regarding the cytotoxicity of AgNPS [1]. Nanoparticles, once they enter the blood stream, first come in contact with vascular endothelial cells, and then liver becomes one of the main target organ of their cytotoxicity [18]. The toxicity of AgNPs to humans has also been recognized, with industrial norms of exposure proposed [19].

The aim of this study was to evaluate Ag concentration in explanted, cirrhotic livers obtained from patients with the history of long-lasting disease and medical treatment. The levels of essential metals, iron, zinc, copper, and of gamma-glutamyl transpeptidase (GGTP), the marker of liver disease, were also evaluated.

## 2. Materials and Methods

### 2.1. Determination of Metal Concentrations in Human Livers and GGTP in Serum

The liver samples were obtained during “back table procedure” while collecting minimal portions of tissue for routine control purposes prior to organ implantation, during liver transplant surgery performed in the Department of General, Transplant, and Liver Surgery, Medical University of Warsaw. The samples were collected from recipient livers (recipients, investigated group with cirrhotic livers, *n* = 44/33 for GGTP), including male (*n* = 25/18) and female (*n* = 19/15) aged 20–61 years (median 45 years), and from donor livers (donors, control group with normal livers, *n* = 28/17 for GGTP), including male (*n* = 13/6) and female (*n* = 15/11) aged 20–68 years (median 48 years). Cirrhosis resulted from Primary Sclerosing Cholangitis (PSC), *n* = 12; Wilson’s disease (WD), *n* = 8; Hepatitis C (HCV), *n* = 16; Hepatitis B (HBV), *n* = 2; Alcoholic disease, *n* = 6.

Serum concentration of GGTP is a diagnostic marker of liver disease. It was included in routine liver test panel in the Recipients group and was also obtained for some Donors. Biochemical tests were performed in the laboratory of Central Clinical Hospital in Warsaw using standard methods.

The precisely weighed liver samples were stored at −80 °C until wet mineralization was performed using concentrated HNO_3_ with addition of concentrated H_2_O_2_ in a microwave oven. The samples (100–500 µL, depending on the liver sample size) were added to 5 mL of internal laboratory standards and measured using Inductively Coupled Plasma Mass Spectrometry (ICP-MS) on an Elan DRC II instrument (Perkin Elmer). The mineralization and ICP-MS determinations were performed in the Central Chemical Laboratory of Polish Geological Institute, National Research Institute, Warsaw, Poland. Calibration solutions and control samples were prepared from certified reference materials. Internal standards (Re and Rh, 10 µg/L) were applied to minimize the matrix effects. The quality of measurements was assured by analyzing blind calibration samples and control samples at the beginning of the series, after every tenth sample measurements and at the end of the series. Every tenth liver sample was reanalyzed as well. The detection limit values were assigned to those silver determinations that yielded the result below the detection limit of the method (0.25 ng of silver in the 500 µL sample).

### 2.2. Statistical Analysis

All statistical analyses and data presentations were done with the aid of R package [20]. As, according to the Shapiro–Wilk test, some metal concentration distributions were found to be non-Gaussian, the nonparametric Mann–Whitney–Wilcoxon test was used to estimate the *p*-value associated with the null hypothesis that the two tested distributions of studied metals were equal. Analogously, Kruskal–Wallis test with the post hoc Dunn test were used to compare metal distributions in more than two groups. The correlations between liver silver and copper concentrations and serum concentrations of GGTP and correlations between liver silver and age and gender of donors and recipients were evaluated using the test for correlation between paired samples with Spearman’s ρ correlation coefficient. In all analyses, the results were considered significant if *p*-value was <0.05. Raw data for the analysis are provided in Table S1 of Supplementary Material.

## 3. Results

**Figure 1:**
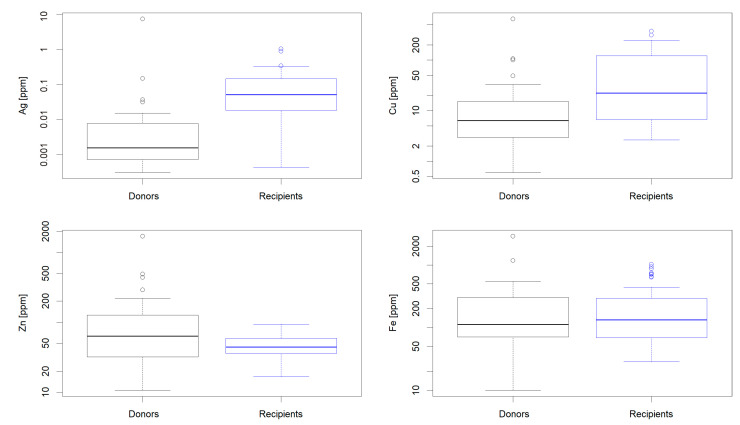
Ag, Cu, Zn, and Fe concentration in donors (black) and recipients (blue) livers. These concentrations are compared with rectangle boxes corresponding to the 1st and 3rd quartiles, thick lines denote the median, thin horizontal lines mark the min–max range, and circles represent outliers according to Grubb’s test. The value of 0.25 ng per liver sample was assigned to samples with the Ag signal below the detection limit.

The concentration box plots in ppm (μg of metal per g of wet liver sample) for Ag, Fe, Zn, and Cu in recipients and donors are shown in Figure 1. Ag was detected in 38 recipients and seven donors, what comprised 87% and 25% of all included into the study, respectively. For the remaining 13% of recipients and 75% of donors the Ag content in the liver samples was found below the detection limit of 0.25 ng. In order to facilitate the statistical analyses, these samples were assigned the maximum undetectable values, obtained as the detection limit divided by the mass of the particular liver sample (See Table S1 for details). This treatment assured that no false positive correlation could be obtained. For some analyses the 0 values were assigned to these samples. Such treatment did not change the outcome of analysis (see Table 1). Other studied metals were detected in all tested samples. The median of Ag concentration in recipients was 0.049 ppm (quartiles 0.019; 0.142 ppm), while in donors, after adjustment for the detection limit for the whole sample (0.25 ng), it was 0.0016 ppm (quartiles 0.0007; 0.0072 ppm). Ag concentrations in recipients and donors differed substantially (*p* < 10^−6^), according to the nonparametric Mann–Whitney–Wilcoxon test.

The odds ratio (OR) of 4.1 (95% C.I. 2.0; 8.1; *p* < 10^−4^) was obtained from these data, indicating that the 10-fold increase in silver concentrations is associated with 4-fold increase in risk in the liver disease. The concentration of Cu was significantly lower in donors comparing to recipients (*p* < 0.003). No significant differences of distributions were observed between the investigated groups for essential metals, Fe and Zn (0.12 and 0.76, respectively).

**Figure 2:**
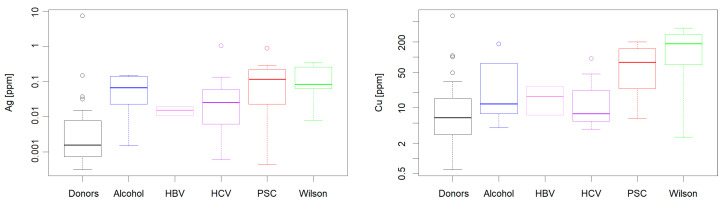
Ag and Cu levels in recipients subdivided with respect to the disease type, compared with the donors. For Ag, individual disease types differ significantly from recipients, but do not differ among themselves. For copper, Primary Sclerosing Cholangitis (PSC) and Wilson’s disease (WD) differ significantly from both other recipients and donors. The circles represent outliers according to Grubb’s test. The corresponding data for Fe and Zn are given in Figure S1. The results of post hoc Dunn test are provided in Table S3.

Figure 2 presents the subdivision of among silver and copper levels into the disease types. The statistics for Figure 1 and Figure 2 are given in Supplementary Material file, Table S2. According to the Kruskal–Wallis test the six groups differed in Ag and Cu concentrations (*p* < 10^−4^ and *p* < 0.002, respectively). Post hoc Dunn test demonstrated that the Ag concentration in liver samples for all groups except of HBV Recipients differed from that of donors, while within the recipients group there were no statistically significant differences between particular diseases (see Table S3). Only the Wilson Disease (WD) and PSC groups differed from donors with respect to the Cu level (Table S3). The corresponding data for Fe and Zn are provided in Figure S1. There were no statistically significant differences in distributions of these essential metals (according to Kruskal–Wallis test *p* = 0.07 and 0.34, respectively).

**Figure 3:**
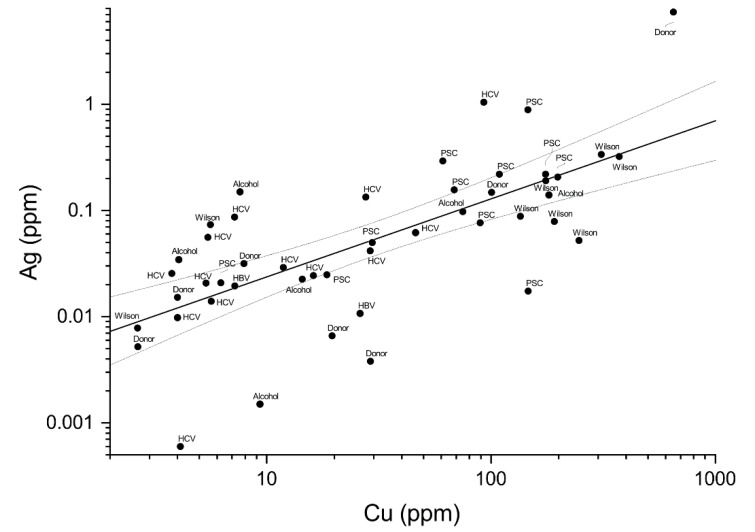
Correlation between silver and copper concentrations in livers of study subjects, with their disease status labeled in the plot. Individuals with silver levels below the detection limit were not included. Thick line represents the log–log correlation; thin lines denote 95% confidence bands.

**Figure 4:**
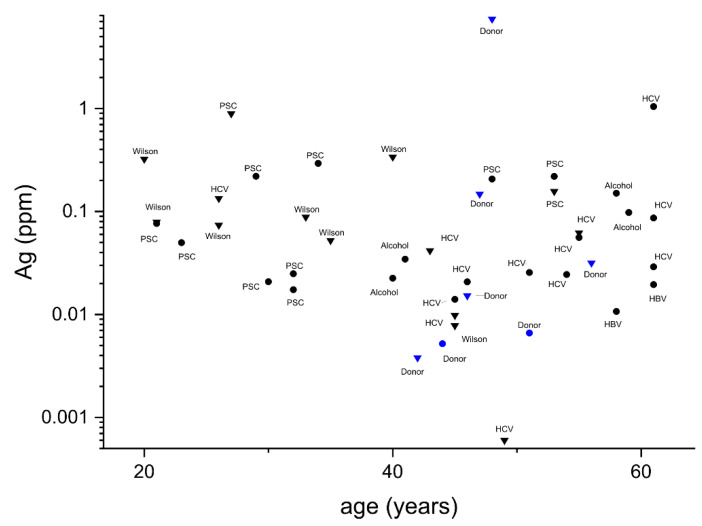
The plot of silver concentrations in livers of study subjects versus age, with their disease status and gender (triangles—male; circles—female) labeled in the plot. Individuals with silver levels below the detection limit were not included in the plot. No correlation between these parameters was found (see Table 1 for details).

The overall relationship between the measured Ag and Cu levels in tested livers is presented in Figure 3. It should be noted that due to non-normal distributions in both groups, necessitating the nonparametric tests, the Pearson’s coefficient could not be calculated. Anyway, the quantitative correlation (i.e., Spearman ρ) between these two metals is evident. The absence of relationship between the Ag level and age and gender in both groups is illustrated in Figure 4 and confirmed by Spearman and Mann–Whitney–Wilcoxon tests, respectively.

## 4. Discussion

Our investigations demonstrated the detectable presence of Ag in livers of most members of the recipient group and in a minority of donors, thus confirming that human liver could accumulate silver. Previous determinations of liver silver in unexposed controls yielded average results around or below 0.05 ppm of tissue wet weight, but a low number of studies and large variability of other factors precludes more detailed dissection of the results [21,22,23,24]. In the most recent of these studies, hepatitis B patients were investigated, but only the average value of 0.045 ± 0.030 ppm was given and a control group was not included [24]. The level of silver in these patients was correlated to other heavy and anthropogenic metals and ascribed to fish consumption.

About 100-fold higher values were historically recorded in patients with amalgam tooth fillings with a positive correlation between the number of amalgam-containing teeth and the Ag level [25]. In these cases, however, the parallel exposure to highly toxic mercury confounded any investigations of health effects specific to silver in those exposures. A single measurement of a severe burn victim treated with silver sulfadiazine cream yielded a still higher value of 14 ppm [23].

The medical records of Recipients do not include systematical treatment with silver-containing medications, such as wound pads, therefore the possible sources of silver are limited to consumer goods enriched or coated with AgNPs. The usually considered routes of human exposure to AgNPs are ingestion, inhalation, or dermal contact [16]. However, we showed that the median concentration of Ag in cirrhotic livers was about 30-fold higher compared to normal livers, but this high concentration was not correlated with a specific pathology causing cirrhosis.

This finding suggests that medical devices or materials used in health care facilities as a possible source of silver in recipients’ livers. This group consisted of terminally ill people who underwent long-term clinical treatment. In such case a non-oral route of administration should be considered, due to the fact that some of such devices can directly release Ag from antimicrobial coatings into lymph and blood vessels.

Despite the wide use of silver in medicine, very few studies were performed to evaluate the absorption rate of silver into bloodstream. Brouillard et al. showed that serum levels of silver were elevated in patients with chronic wounds even after one month of treatment with silver-containing dressings [26]. Predictive factors for systemic silver absorption were wound area, anemia, and malnutrition. The latter two factors always accompany cirrhosis. They also showed slow elimination of silver from the organism. Similar results were obtained by Wang et al. in pediatric patients treated with Anticoat [27]. They also showed in a porcine model with 2% total body surface area burns that silver, after application of Anticoat accumulates in liver, kidney, heart, and brain.

Studies on bioavailability, biodistribution, and kinetics of silver, mostly in rodents, depended on the chemical form of absorbed silver, e.g., AgNPs or ions, route of administration, and dose. In the case of AgNPs, the size of particles and the kind of coating also played a role. The data from different studies with different study protocols are thus difficult to compare with each other. Clearly, however, the oral administration of AgNPs yielded significantly lower Ag serum concentration than intravenous administration, which can be partially explained by low Ag absorption rate from gastrointestinal tract, and feces as the main route of elimination [28,29]. Either administration route resulted in translocation of AgNPs from blood into organs, preferentially into the liver. However, oral administration of either AgNPs or silver acetate resulted in nanogranules containing silver being detected in the basal lamina of ileal epithelium and in lysosomes of macrophages within the lamina propria [28,29,30,31,32,33,34]. Therefore, in order to estimate the impact of AgNPs on human health, it is of paramount importance to consider both the low dose lifelong dietary exposure to Ag and the direct exposure of circulating blood and lymph to AgNP-coated medical devices.

Cu concentration was increased in recipients, with a strong positive correlation with Ag (*p* < 10^−6^). A similar correlation was also found in those donors that exhibited measurable Ag (*p* < 0.004). In contrast, concentrations of Fe and Zn differed neither between study groups nor among individual disease types, indicating that the general metal metabolism was not affected. No correlation between silver and copper in liver tissue was found in the Turkish hepatitis B study, where silver exposure, presumably to ionic silver, occurred via food [24].

Copper is toxic to hepatic cells, by inducing complex phenomena including oxidative stress, disruption of intracellular zinc distribution, and induction of apoptosis [35,36,37]. Copper transporter 1 (Ctr1), the major transmembrane copper transporter in eukaryotic cells, is highly selective against other metal ions, but can transport Ag(I) ions [38]. Consequently, the exposure of cells to Ag(I) was shown to inhibit the cellular copper entry by competition at Ctr1 [39]. We, however, recorded an opposite effect in livers exposed to silver. A hypothetical scenario of molecular events in hepatocytes could consist of massive silver entry into the cells by AgNPs endocytosis, as demonstrated [13], followed by their oxidative dissolution to ionic Ag(I) in lysosomes and the cytosol [11]. This could result in Ag(I) interfering with copper export systems as a mechanism of copper accumulation in liver upon silver exposure.

ATP7B was demonstrated to detoxify human cells from exposure to ionic silver [40]. The ion transport by ATP7B is energy (ATP)-consuming and saturatable [41]. Therefore, one can easily envisage that a huge amount of Ag(I) ions delivered by AgNPs dissolution can inhibit Cu(I) export by ATP7B. Deleterious mutations in ATP7B are responsible for Wilson’s disease (WD), the condition when copper is accumulated in tissues, primarily liver, causing oxidative stress [42]. Indeed, eight recipients suffered from WD and had elevated liver copper, and the silver levels in their livers were among the highest. If Ag(I) indeed blocked or slowed down Cu(I) export from hepatocytes via ATP7B, then one might consider the AgNPs effect on liver as sort of induced WD condition. A support for this supposition can be found in three independent studies of hepatocyte exposure to AgNPs, which found physiological and proteomic markers of oxidative stress [43,44,45]. Other possibilities include intracellular retention of both copper and silver by binding to copper chaperones and metallothioneins, and intracellular redistribution, e.g., to organelles via Copper transporter 2 Ctr2 [46].

The levels of GGTP were not correlated with either Cu or Ag levels in donor livers, which is consistent with the good conditions of the donated organs. In recipients, the correlation between Cu alone and GGTP was not significant, despite the participation of WD patients, but such significance was detected in Donors and Recipients taken together. On the other hand, the positive correlation between the Ag burden and GGTP in recipients was significant (Table 1). Donor livers formally did not exhibit a significant relationship between Ag and GGTP, but the number of donors with measurable Ag level was too low to draw conclusions. In general, elevated GGTP is considered to be a sign of alcohol disease or biliary obstruction, but a recent review paper indicated the relationship between the elevated GGTP and cellular oxidative stress [47]. This correlation supports the notion that the Ag exposure detected in this study may pose a significant health hazard, by contributing to liver damage caused by the basic disease in recipients. In this context, the apparent weak correlation between GGTP and Cu was probably coincident, due to the correlation of both GGTP and Cu levels with Ag.

A previous in vitro study revealed that the mechanism of induction of AgNPs cytotoxicity in cultured liver cells included autophagy and lysosomal membrane permeabilization, resulting in NOD-, LRR- and pyrin domain-containing protein 3 NLRP3 inflammasome-dependent caspase-1 activation [48]. Toxic effects of silver were also attributed to oxidative stress, reactive oxygen species generation, and mitochondrial and DNA damage [1]. Animal studies revealed that AgNPs toxicity is dose- and size-dependent [31,34]. Transcriptomic analysis of rabbit tissue after AgNPs exposure indicated potential carcinogenicity of silver in terms of continuous genomic instability. A total of 244 genes were differentially expressed 28 days after a single dose of AgNPs. High correlation of these genes with inflammation, hepatotoxicity, and cancer was confirmed [2]. Histological examination of rat liver after 8 weeks of oral treatment with AgNPs in the dose of 10 mg/kg showed distortion in hepatocellular arrangement, nuclear condensation and pyknosis, and areas of vascular changes suggesting loss of liver architectural support or fibrosis [49]. These data revealed that silver exposure in the form of nanoparticles could provoke multifaceted, continued, and prolonged damage of liver tissue structure and function. Furthermore, very recent data indicate that even apparently non-toxic exposures to AgNPs may impair liver function, by specific inhibition of nuclear receptors, zinc finger effectors of hormonal activity [50]. Ag(I) ions were recently shown to easily remove Zn(II) ions from zinc fingers, providing molecular background for this process, and indicating that AgNPs may impair zinc-specific pathways as well, even without affecting the bulk cellular zinc [15].

In conclusion, our results indicate that human liver accumulates silver, especially in patients with long medical treatment history and potentially increased exposure to AgNPs present in standard medical devices, such as catheters coming into direct contact with body fluids. While we could not exclude that cirrhosis itself enhances silver accumulation in liver, it could augment the disease and aggravate clinical outcomes. Correlation between silver and copper concentration in cirrhotic liver could indicate a potentially synergistic adverse effect on liver function. Taking into account the widespread and increasing use of AgNPs, their potential hepatotoxicity becomes an urgent issue in public health.

## Figures and Tables

**Table 1:** Pairwise Spearman correlation ρ coefficient and associated p-value calculated for serum gamma-glutamyl transpeptidase (GGTP), liver copper and silver, age and gender in Donors and Recipients.

Groups	ρ (*p*-Value)		
	Recipients	Donors	All
Ag vs. Cu	0.67 (1.5 × 10^−6^)	0.53 (0.004)	0.69 (2 × 10^−11^)
Ag vs. GGTP	0.37 (0.03)	−0.04 (0.85)	0.55 (4 × 10^−5^)
Cu vs. GGTP	0.12 (0.66)	0.20 (0.26)	0.38 (0.007)
Ag vs. age Undetectable Ag omitted	−0.16 (0.31)	0.61 (0.17)	−0.13 (0.38)
Ag vs. gender Undetectable Ag omitted Wilcoxon stat. (p)	W = 222 (0.35)	6 (1)	292 (0.60)

## Data Availability

The data presented in this study are available in the supplementary material to the present article.

## Supplementary Materials

Supplementary Materials can be found at https://www.mdpi.com/1422-0067/22/4/1782/s1. Table S1. Raw data used for statistical analysis. Table S2. Statistical data on metal contents (wet weight) in Donors and Recipients (total and subdivided according to disease), used to generate Figure 1 and Figure 2 and Figure S1. Table S3. The results of post hoc Dunn’s test for Ag and Cu levels in Donors and Recipients subdivided according to liver disease. The *p*-values below the rejection limit (≤α/2 = 0.025) of the null hypothesis of nonsignificance of differences are marked in bold red. Figure S1. Zn and Fe levels in recipients subdivided with respect to the disease type compared with the donors. There is no statistical significance between study groups with respect to either metal. The circles represent outliers according to Grubb’s test.

## Abbreviations

AgNPs	silver nanoparticles
PSC	Primary Sclerosing Cholangitis
WB	Wilson’s disease
HBV	Hepatitis B
HCV	Hepatitis C
GGTP	Gamma-glutamyltranspeptidase
ICP-MS	Inductively Coupled Plasma Mass Spectrometry
Ctr1	Copper transporter 1
Ctr2	Copper transporter 2
NLPR3	NOD-, LRR- and pyrin domain-containing protein 3
