# Supplementary material for: Cirrhotic Liver of Liver Transplant Recipients Accumulate Silver and Co-Accumulate Copper
Source: Int J Mol Sci. 2021 Feb 11;22(4):1782. doi: 10.3390/ijms22041782 (PMC7916850; doi:10.3390/ijms22041782)
Supplement: Supplementary file 1 [file ijms-22-01782-s001.pdf]

Supplementary Material

Table S1. Raw data used for statistical analysis. The X symbol denotes silver readouts below the detection limit.

Sample code	Diagnosis	Gender	Age	Fe (μg/L)	Cu (μg/L)	Zn (μg/L)	Ag (μg/L)	GGTP (IU/L)	Sample mass (g)
C005	PSC	F	46	28.66	8.23	76.52	X	120	0.227
C007	PSC	M	23	224.46	29.54	57.17	0.0498		0.166
C008	WILSON	F	21	206.97	191.23	42.24	0.079	110	0.082
C012	ALCOHOL	M	40	303.31	14.38	34.83	0.0225	154	0.445
C013	ALCOHOL	M	41	98.3	4.05	37.26	0.0344	589	0.095
C016	HCV	M	61	942.59	11.83	32.2	0.029	51	0.155
C018	ALCOHOL	M	59	641.78	74.86	46.41	0.0976		0.085
C019	HCV	F	55	1035.5	46.11	41.39	0.062	117	0.214
C020	WILSON	F	45	255.33	2.65	16.65	0.0078	108	0.224
C023	WILSON	F	35	230.94	246.58	60.31	0.0523	102	0.162
C024	PSC	M	21	43.96	89.47	69.68	0.0764	468	0.304
C025	HCV	F	45	70.45	4	45.73	0.0098	43	0.358
C027	HCV	M	61	105.69	92.83	29.18	1.0445	124	0.444
C031	PSC	F	53	46.09	68.44	36.11	0.1568	262	0.053
C032	HCV	M	46	123.15	5.36	47.96	0.0207	170	0.277
C034	PSC	M	53	123.96	174.75	49.95	0.2193	140	0.529
C036	HCV	M	61	329.66	7.17	89.71	0.0865	126	0.023
C037	WILSON	F	40	213.07	309.8	72.95	0.3378		0.190
C039	PSC	F	27	51	145.86	48	0.89	236	0.389
C045	HCV	F	43	63.29	28.93	29.46	0.0417	129	0.193
C046	ALCOHOL	M	58	104.38	7.6	24.96	0.15	45	0.234
C047	WILSON	F	26	76.08	5.6	30.94	0.0736	185	0.242
C050	WILSON	F	40	109.9	175.09	42.49	0.1913		0.393
C051	PSC	M	29	152.19	108.85	54.76	0.2194	553	0.013
C052	HCV	M	55	104.09	5.46	66.91	0.0558	75	0.238
C053	PSC	M	34	73.54	60.83	57.84	0.2926	319	0.238
C055	HCV	F	52	61.02	5.97	35.77	X	57	0.040
C056	HCV	F	46	873.54	8.43	49.3	X	73	0.041
C058	HCV	M	51	163.16	3.77	21.52	0.0255	51	0.415
C060	PSC	M	32	72.68	18.52	63.32	0.0248	41	0.483
C067	PSC	M	32	34.86	146.28	41.03	0.0174	106	0.048
C068	WILSON	F	33	440.06	134.94	47.69	0.0881		0.210
C074	HCV	F	49	705.7	4.12	29.69	0.0006	110	0.297
C075	HCV	M	45	65.97	5.65	41.92	0.014		0.297
C076	HCV	M	54	721.18	16.15	27.75	0.0244	141	0.349

C084	ALCOHOL	M	48	284.62	9.33	84.73	0.0015		0.140
C089	WILSON	F	20	67.43	372.29	93.55	0.3219		0.274
C094	HCV	F	56	148.38	8.08	56.89	X	52	0.086
C095	ALCOHOL	M	45	750.77	181	65.34	0.1398		0.214
C100	PSC	M	30	249.69	6.24	63.23	0.0208	167	0.181
C101	HCV	F	26	140.61	27.64	41.37	0.1338	82	0.271
C102	HBV	M	61	51.11	7.2	36.12	0.0195		0.211
C103	PSC	M	48	57.24	198.19	37.97	0.2065	73	0.038
C105	HBV	M	58	652.06	26.03	41.85	0.0107		0.140
									--
Z04	donor	F	24	78.73	2.35	24.09	X	19	0.200
Z05	donor	M	48	61.66	9.69	110.55	X		0.069
Z06	donor	F	38	66.86	7.29	60.41	X		0.174
Z07	donor	F	63	292.54	2.57	41.7	X		0.015
Z08	donor	M	57	206.5	48.37	436.47	X		0.011
Z09	donor	M	49	1185.66	32.68	488.29	X		0.003
Z10	donor	F	20	537.24	4.03	54.59	X	13	0.025
Z11	donor	M	44	110.69	2.66	25.85	0.0052		0.162
Z13	donor	F	39	97.78	0.6	10.7	X	20	0.139
Z14	donor	M	51	184.5	19.53	218.94	0.0066		0.092
Z15	donor	F	30	103.4	6.12	140.6	X	12	0.079
Z16	donor	M	48	275.31	649.12	204.98	7.4003	18	0.021
Z19	donor	F	46	113.86	4	39.96	0.0152		0.062
Z20	donor	M	50	469.91	5.64	92.96	X	26	0.102
Z21	donor	M	53	80.2	4.26	34.58	X	44	0.164
Z22	donor	F	50	20.58	1.23	13.78	X	8	0.113
Z23	donor	F	42	456.09	29	290.12	0.0038	44	0.041
Z24	donor	F	43	10.07	3.22	32.8	X	6	0.137
Z26	donor	F	52	156.64	6.3	66.39	X	124	0.189
Z27	donor	F	54	74.84	1.49	17.93	X	43	0.137
Z28	donor	M	68	84.33	6.41	112.77	X		0.025
Z29	donor	M	27	39.78	1.25	20.08	X	18	0.128
Z30	donor	M	49	53.03	9.91	110.3	X		0.060
Z31	donor	M	37	2908.87	107	1716.8	X	16.8	0.007
Z32	donor	F	54	314.13	10.69	30.93	X	10	0.132
Z33	donor	M	31	130.49	7.45	91.17	X	12	0.323
Z34	donor	F	56	547.71	7.91	45.02	0.0316	51	0.070
C059	donor	F	47	45.92	100.53	66.98	0.1477		0.308

Table S2. Statistical data on metal contents (wet weight) in Donors and Recipients (total and subdivided according to disease), used to generate Figures 1, 2 and S1.

Group	#persons	min	Q1	median	Q3	max
			**Ag [ppm]**			
**Donors**	28	0.0003	0.0007	0.002	0.007	7.40
**Recipients**	44	0.0004	0.019	0.051	0.142	1.04
Alcohol	6	0.0015	0.026	0.066	0.129	0.15
HBV	2	0.0107	0.013	0.015	0.017	0.02
HCV	16	0.0006	0.008	0.025	0.057	1.05
PSC	12	0.0004	0.024	0.117	0.219	0.89
WD	8	0.0078	0.068	0.084	0.224	0.34
			**Cu [ppm]**			
**Donors**	28	0.6	3.1	6.4	13	649
**Recipients**	44	2.7	6.9	22	115	372
Alcohol	6	4.1	8.0	12	60	181
HBV	2	7.2	12	17	21	26
HCV	16	3.8	5.4	7.6	19	93
PSC	12	6.2	27	79	146	198
WD	8	2.7	103	183	262	372
			**Fe [ppm]**			
**Donors**	28	10	73	112	298	2909
**Recipients**	44	29	70	132	289	1036
Alcohol	6	98	149	294	557	751
HBV	2	51	201	352	502	652
HCV	16	61	96	144	710	1036
PSC	12	29	46	65	131	250
WD	8	67	101	210	237	440
			**Zn [ppm]**			
**Donors**	28	11	32	63	120	1717
**Recipients**	44	17	36	44	58	94
Alcohol	6	25	35	42	61	85
HBV	2	36	38	39	40	42
HCV	16	22	30	41	48	90
PSC	12	36	46	56	63	77
WD	8	17	39	45	63	94

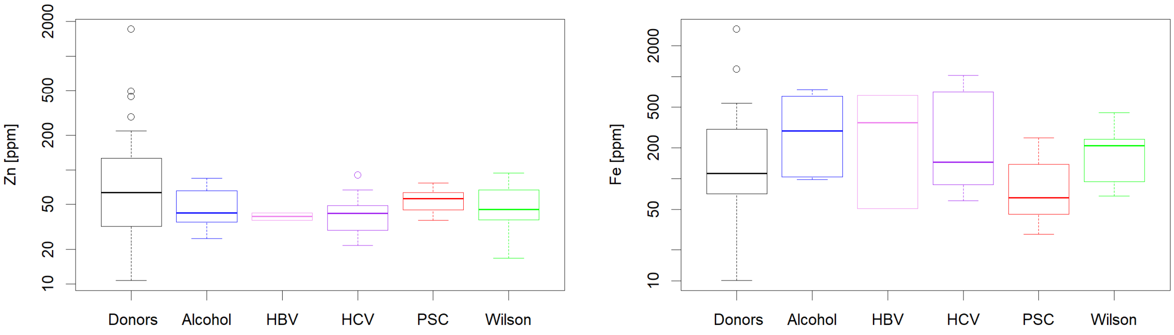

Figure S1. Zn and Fe levels in Recipients subdivided with respect to the disease type. compared with the Donors. There is no statistical significance between study groups with respect to either metal. The circles represent outliers according to Grubb's test.

Table S3. The results of post-hoc Dunn's test for Ag and Cu levels in Donors and Recipients subdivided according to liver disease. The p values below the rejection limit (≤α/2 = 0.025) of the null hypothesis of nonsignificance of differences are marked in bold red.

**Ag**						
Kruskal-Wallis		27.0105,	df	5,	p-value	0
	Donors	Alcohol	HBV	HCV	PSC	
Alcohol	-2.61					
	**0.005**					
HBV	-0.76	0.76				
	0.225	0.223				
HCV	-2.55	0.79	-0.33			
	**0.006**	0.216	0.372			
PSC	-4.01	-0.42	-1.09	-1.54		
	**0.000**	0.337	0.138	0.062		
WD	-3.93	-0.74	-1.29	-1.79	-0.41	
	**0.000**	0.229	0.098	0.037	0.339	
**Cu**						
Kruskal-Wallis		19.3602,	df	5,	p-value	0
	Donors	Alcohol	HBV	HCV	PSC	
Alcohol	-1.42					
	0.078					
HBV	-0.61	0.23				
	0.271	0.408				
HCV	-0.55	0.97	0.36			
	0.291	0.166	0.358			
PSC	-3.51	-1.15	-1.00	-2.72		
	**0.000**	0.126	0.158	**0.003**		
WD	-3.15	-1.16	-1.04	-2.52	-0.12	
	**0.001**	0.123	0.150	**0.006**	0.453	
